# Genomic Insights Into the Multiple Factors Controlling Abdominal Fat Deposition in a Chicken Model

**DOI:** 10.3389/fgene.2018.00262

**Published:** 2018-07-19

**Authors:** Bahareldin A. Abdalla, Jie Chen, Qinghua Nie, Xiquan Zhang

**Affiliations:** ^1^Department of Animal Genetics, Breeding and Reproduction, College of Animal Science, South China Agricultural University, Guangzhou, China; ^2^National-Local Joint Engineering Research Center for Livestock Breeding, The Key Lab of Chicken Genetics, Breeding and Reproduction, Ministry of Agriculture, Guangzhou, China; ^3^Guangdong Provincial Key Lab of Agro-Animal Genomics and Molecular Breeding, The Key Lab of Chicken Genetics, Breeding and Reproduction, Ministry of Agriculture, Guangzhou, China

**Keywords:** abdominal fat deposition, QTL, SNP, gene expression studies, miRNAs, lncRNAs, epigenetic alterations

## Abstract

Genetic selection for an increased growth rate in meat-type chickens has been accompanied by excessive fat accumulation particularly in abdominal cavity. These progressed to indirect and often unhealthy effects on meat quality properties and increased feed cost. Advances in genomics technology over recent years have led to the surprising discoveries that the genome is more complex than previously thought. Studies have identified multiple-genetic factors associated with abdominal fat deposition. Meanwhile, the obesity epidemic has focused attention on adipose tissue and the development of adipocytes. The aim of this review is to summarize the current understanding of genetic/epigenetic factors associated with abdominal fat deposition, or as it relates to the proliferation and differentiation of preadipocytes in chicken. The results discussed here have been identified by different genomic approaches, such as QTL-based studies, the candidate gene approach, epistatic interaction, copy number variation, single-nucleotide polymorphism screening, selection signature analysis, genome-wide association studies, RNA sequencing, and bisulfite sequencing. The studies mentioned in this review have described multiple-genetic factors involved in an abdominal fat deposition. Therefore, it is inevitable to further study the multiple-genetic factors in-depth to develop novel molecular markers or potential targets, which will provide promising applications for reducing abdominal fat deposition in meat-type chicken.

## Introduction

Adipose tissue is an essential endocrine organ producing hormones and other substances that can deeply affect our health (Butterwith, 1997; Gregoire et al., 1998; Kershaw and Flier, 2004; Resnyk et al., 2015). Abdominal fat and subcutaneous fat are major parts of adipose tissue but researchers hint that abdominal fat cell is more biologically active (Ibrahim, 2010). In addition, abdominal fat depositions are of particular concern because it's a key factor in a variety of health problems. Chicken abdominal fat deposition is regulated by genetic, endocrine hormones, environmental factors and multiple behavioral (Cahaner and Nitsan, 1985; Fouad and Elsenousey, 2014; Resnyk et al., 2015). Chicken abdominal fat has a higher heritability rate (0.82) than that of live body weight (0.55) and body parts; thighs (0.31), drumsticks (0.51), and breast muscle (0.55) at the age of selection (Cahaner and Nitsan, 1985). Abdominal fat accumulation needs more energy (wastes feed and increases costs to farmers) than does lean tissue accumulation and farmers are interested in reducing cost by increasing system efficiency. There is a positive correlation between abdominal fat traits and body weight in chicken (Zerehdaran et al., 2004). High-fat content in poultry can be a risk to human health (Milićević et al., 2014). To date, high-abdominal adipose accumulation in a commercial broiler is an obstacle to the profitable farming (Chambers, 1990; Zhou et al., 2006; Sun Y. et al., 2013; Zhang et al., 2017). In human, studies have shown that excessive abdominal fat deposition is associated with metabolic syndrome (Björntorp, 1992; Després, 2006; Alvehus et al., 2010). Although intensive selection in chickens has increased daily growth rate and body weight and reduced market age, commercial broilers have increased the tendency toward physiological disorders, such as obesity (Li et al., 2003). Obesity is a serious public health problem around the world that increases the risk of some common diseases, such as type 2 diabetes, cardiovascular disease, metabolic syndrome, hypertension, and a few types of cancer (Kopelman, 2000; Alvehus et al., 2010), and it arises from a complex interaction between genetic and environmental factors (Norman et al., 1997; Romao and Roth, 2008). Overweight and obesity are defined as abnormal or excessive fat deposition that damages health. However, the genetic and molecular mechanisms that link to obesity are largely not known. The genes or molecules that regulating abdominal fat deposition or abdominal adipose tissue development can be identified by many different genomic approaches (Tatsuda and Fujinaka, 2001; Abasht and Lamont, 2007; Huang et al., 2015a; Ouyang et al., 2016; Jin et al., 2017; Zhang et al., 2017). Comparison studies between lean and fat chickens in abdominal fat can provide useful information about the underlying genetic variation in the trait.

The purpose of this review paper is to summarize the current understanding of genetic/epigenetic factors associated with abdominal fat deposition, or as it relates to proliferation and differentiation of preadipocyte in chicken. Researchers have investigated the abdominal fat deposition in chicken using different genomic approaches, such as quantitative trait locus (QTL) analysis, the candidate gene approach, epistatic interaction, copy number variation (CNV), single-nucleotide polymorphism (SNP) screening, genome-wide association studies (GWAS), selection signature analysis, gene expression studies (microarray and RNA sequencing), microRNA (miRNA) and long non-coding RNA (lncRNA) regulation, and bisulfite sequencing analysis.

### Chicken as a research model

Chicken has been utilized as a good animal model to study basic mechanisms of adipogenesis, embryonic development, immune function, nutrition, endocrine function, and cancer (Butterwith, 1997; Hillier et al., 2004; Bader et al., 2006; Fouad and Elsenousey, 2014; Jun et al., 2014; Gan et al., 2015; Li et al., 2017). Chicken embryos are a unique model that overcomes many limitations for investigating cancer biology (*in vivo* method). Commercial broilers can be used as a good biomedical model to study obesity or obesity-related disease. In fact, there are many good reasons that make the chicken a useful model for studying adipogenesis or obesity. For example, the good isolated chick preadipocyte does not contain any other contaminating cell types, which can be an obstacle in cell isolation from other species (Butterwith, 1997). An *in vitro* differentiation of these cells can be achieved by a similar method of hormonal induction to that used for rodents and human (Ramsay and Rosebrough, 2003). In addition, abdominal fat weight (AFW) or abdominal fat percentage (AFP) in chicken can be measured directly (but not the human abdominal fat), and experiments can be designed to identify genetic variants associated with abdominal fat traits. The results from this type of experiment not only provide useful comparative information for human obesity studies but also lead to the genetic improvement for reducing abdominal fat deposition in chicken. Moreover, there are fundamental similarities between chicken and human genomes. For example, Hillier et al. (2004) reported that the chicken genome is significantly smaller in size than the human genome, but almost the same number of genes. This explains that chicken genome has a substantial reduction in the DNA duplications, DNA repeat sequences, and fewer pseudogenes. About 60% of chicken genes correspond to almost the same as a human gene. Chicken genes responsible for the fundamental structure and function of the cells displayed more similarity in the sequence with human genes than did those involved in reproduction, host defense mechanisms and environmental adaptation. Sequence alignments of chicken and human genes show that about 2,000 human genes may truly start at different locations than previously thought. The detection of these (true) start locations, which seem to lie within the previously hypothesized gene boundaries, may help for understanding human diseases and the design and development of new therapies. Chickens possess interleukin-26 (IL-26) gene, a protein regulating immune response. This IL-26 was found only in human. The finding indicates that the chicken can now serve as a model organism in which studies can examine IL-26 function. Transgenic chicken, gene editing etc. methods have now been established in chicken (Cooper et al., 2017), this can give many advantages same as mice and rats as an avian model for future studies of complex human diseases.

### Adipose tissues

Mammalian adipose tissues can be anatomically and functionally distinct into white adipose tissue (WAT) and brown adipose tissue (BAT). Recent studies have identified a new distinct type of thermogenic adipocyte interspersed within WAT, called beige cells or brite cells. Beige and brown adipocytes seem to be functionally similar (Shabalina et al., 2013). White adipocyte contains a single large lipid droplet, but brown adipocyte has many small lipid droplets, a large number of iron-containing mitochondria, and more capillaries than white adipocyte. The high iron content gives brown adipocyte its identity; brown color (Enerbäck, 2009). WAT plays a key role in energy homeostasis, stores excess energy in the form of triglycerides. BAT takes calories from WAT and burns it for producing heat (Sarjeant and Stephens, 2012). In human, BAT is abundant in newborns, which helps them keep warm by thermogenesis mediated by uncoupling protein-1 (Cypess et al., 2009). Previously, BAT was believed to be almost absent and without physiological role in human adults (Cannon and Nedergaard, 2004). It is currently accepted that BAT is existed in posterior regions of the neck, thorax, and abdomen of lean and obese adult humans, and may regulate energy metabolism, cold sensitivity, and body-weight gain (Leitner et al., 2017; Nedergaard and Cannon, 2017). Similar to white adipocytes, brown adipocytes require key transcription factors (such as peroxisome proliferator-activated receptor gamma (PPARγ), CCAAT/enhancer-binding proteins (C/EBPs), signal transducers and activators of transcription (STATs), and Krüppel-like factor (KLF) proteins) that are so important to accelerate adipocyte precursor cell differentiation into mature adipocyte (Sarjeant and Stephens, 2012). The topic has not been thoroughly investigated or being detected in chicken.

### QTL locations controlling abdominal fat deposition

Quantitative trait locus (QTL) is a section of DNA (the locus) for a gene that influences a quantitative trait. Molecular genetic techniques have made it possible to search genomes for QTL. These loci have been identified and mapped on specific chromosomes for many organisms, including livestock such as chicken and cattle. In general, association studies are useful in the investigation of quantitative traits. In chicken, genetic variations in QTLs for many traits have been studied for over 20 years. The strategy of identifying the QTLs or causal genes that are related to abdominal fat traits is a powerful tool to illustrate the genetic control of abdominal fat deposition. Abdominal fat traits have been a focus of QTL mapping investigations and several chromosomes are involved in chicken (Hu et al., 2016). To our knowledge, study by Tatsuda and Fujinaka (2001) is the first study to identify QTLs affecting abdominal fat deposition in chicken. As reviewed in Table 1, these studies have identified significant QTLs affecting abdominal fat deposition in chicken. Large numbers of QTLs controlling abdominal fat deposition are located on chromosome 1. However, comparing with different approaches such as GWAS and RNA-seg, QTLs exhibit some limitations from which the identified QTL sites are generally large and need subsequent fine mapping to distinguish closely linked genes or causal variants with relevance to target traits.

**Table 1:** QTL positions significantly controlling abdominal fat deposition in chicken.

**(cM)**	**Chr**.	**Flanking marker**	**Abdominal fat trait**	**Population or breed/line (age; days)**	**References**
38	7	MCW316-MCW92	AFP	F_2_ Satsumadori × WPR (112 d)	Tatsuda and Fujinaka, 2001
126	1	ADL0188-LEI0068	AFP	F_2_ originated from White leghorn Layer × Broiler cross (63 d)	Ikeobi et al., 2002
40, 50	3	ADL0177-MCW0083	AFW, AFP		
50, 51	5	ROS0013-ADL0292	AFP, AFW		
39, 41	7	LEI0064-ROS0019	AFP, AFW		
127	9	MCW0135-ROS0030	AFP		
17	13	ADL0147-ADL0225	AFW		
0, 0	15	LEI0083-MCW0080	AFW, AFP		
17	28	ROS0095-ADL0299	AFW and AFP		
127	Z	LEI0111-LEI0075	AFW and AFP		
18, 25	1	ADL0160-HUJ0001	AFP, AFW	F_3_ broiler dam lines originated from WPR (70 d)	Jennen et al., 2004
241	1	MCW0058-MCW0101	AFP		
214	1	LEI0174-ADL0361	AFW		
573	1	ADL0350-MCW0107	AFP	(63 d)	
356	2	MCW0264-ADL0164	AFP	(49 d)	
0	3	MCW0037-MCW0148	AFP	(49 d)	
22	4	LEI0063-MCW0098	AFW	(49 d)	
71, 75	4	LEI0076-MCW0276	AFW, AFP	(63 d)	
126	4	LEI0094-LEI0122	AFW	(49 d)	
149	7	MCW0092-MCW0316	AFW	(70 d)	
27	11	ADL0287-ADL0210	AFW	(49 d)	
0	13	MCW0104-MCW0322	AFW	(49 d)	
0	15	MCW0031-MCW0226	AFP	(49 d)	
21	15	LEI0120-MCW0231	AFW	(70 d)	
22	15	LEI0120-MCW0231	AFP	(70 d)	
24	15	LEI0120-MCW0231	AFP	(63 d)	
21	18	ADL0304-MCW0217	AFP	(70 d)	
23	18	ADL0304-MCW0217	AFW	(70 d)	
0	27	MCW0076- MCW0146	AFP	(63 d)	
94	1	LEI0146-LEI0174	AFP	F_2_ broiler × layer (42 d)	Nones et al., 2005
196	1	ADL0020-LEI0160	AFW		
251	1	ADL0148-MCW036	AFP		
155, 168	1	MCW0297-LEI0101	AFP, AFW	F_2_ broiler × broiler (40–42 d)	McElroy et al., 2006
10, 13	2	MCW0205-MCW0082	AFP, AFW		
274	3	MCW0277-MCW0207	AFP		
160	4	LEI0076-MCW0240	AFW, AFP		
64, 76	5	GCT0050-MCW0106	AFP, AFW		
59	6	LEI0192-ADL0230	AFW		
82, 85	7	MCW0201-MCW0236	AFP, AFW		
25, 27	8	ADL0258- ADL0345	AFW, AFP		
449	1	ADL0328-LEI0061	AFP	F2 broiler × broiler (56 d)	Lagarrigue et al., 2006
84	3	MCW0083-HUJ0006			
121	3	HUJ0006-LEI0161			
68	5	MCW0038-MCW0214			
150	5	ADL0233-ADL0298			
32	7	LEI0064-MCW0120			
438	1	LE10162-LE10134	AFW	F_2_ WPR × WPR (56 d)	Park et al., 2006
125	3	MCW0222-MCW0004			
41	7	ADL0169-ADL0279			
69	1	MCW0010-MCW0106	AFP	F_2_ NEAURP (84 d)	Liu et al., 2007
183	1	LEI0068-MCW0297	AFW		
548, 550	1	ADL0328-ROS0025	AFP, AFW		
205	1	ACW0356-LEI0160	AFW	F_2_ WRR × XH (90 d)	Rao et al., 2007
221	1	MCW0112-MCW200	AFP		

### The candidate gene approach of abdominal fat traits

The candidate gene approach is another popular approach for QTL identification. Functions of genes are excellent candidates for linkage relationships with quantitative traits of important traits (Zhu and Zhao, 2007; Bohannon-Stewart et al., 2014). In general, the candidate gene approach has been widely used in the detection of genes responsible for disease and quantitative traits as well as evolutionary genomics, and has revealed important findings. In chickens, this approach has been used for many decades to distinguish genes associated with important traits. A brief summary of some candidate genes involved in abdominal fat deposition in chicken is given in the following lines.

*Adipocyte fatty acid-binding protein* (*A-FABP*; also known as *FABP4*) is a subfamily of the fatty acid-binding proteins (FABPs), and seem to function in fat metabolism. Shi et al. (2010) evaluated the expression of *FABP4* in different tissues and its correlation between expression of fat and lean chickens in abdominal adipose tissue. The protein expression of this gene was expressed only in abdominal adipose tissue and there was no protein band detected in heart, liver, muscle, muscle stomach, spleen, small intestine, lung, and kidney. The *FABP4* messenger RNA (mRNA) expression in fat chicken was lower than that of lean chicken at 2, 3, 4, 6, 7, 9, and 10 weeks (wk) of age, and the protein expression of fat chicken was lower than that of lean chicken at 6 and 10 wk of age. However, *FABP4* may regulate abdominal fat deposition through lipolysis. Another two types of fatty acid-binding protein subfamily named L-FABP (liver fatty acid-binding protein) and liver “basic” fatty acid-binding protein (Lb-FABP) have been investigated their expression in liver between fat and lean male-chickens by Zhang et al. (2013). *L-FABP* mRNA expression in fat birds was higher than that in lean birds at 2, 3, 4, 5, 6, and 10 weeks of age, and *L-BABP* mRNA expression in fat birds was higher than that in lean birds at 1, 2, 3, 4, 8, and 10 wk of age. Western blotting demonstrated that the L-FABP protein level in fat birds was higher than that in lean birds at 3, 5, 6, and 7 wk of age, and L-BABP protein level in fat birds was higher than that in lean birds at 3, 4, 5, and 6 wk of age. However, *L-FABP* and *L-BABP* may regulate abdominal fat deposition through changes in their expression and lipolysis.

The expression of the *high mobility group AT-hook 1* (*HMGA1*) gene has been shown to be significantly associated with relative abdominal fat content and abdominal fat weight (AFW) in broiler (Larkina et al., 2011). This gene shows a significant expression difference in the liver of broiler between high and low abdominal fat groups, which is increased in high abdominal fat group.

The *fat mass and obesity associated* (*FTO)* gene was identified as an important locus harboring common variants impact on obesity predisposition and fat mass (Scuteri et al., 2005; Frayling et al., 2007). The *FTO* SNP is associated with higher body mass index (BMI), as well as weight and abdominal adiposity in human (Pausova et al., 2009). In chicken, *FTO* mRNA expression is highly expressed in abdominal fat at 19 wk of age in male leghorn layer (Wang Y. et al., 2012). Jia et al. (2012) found variations in *FTO* gene associated with growth, body weight and fatness traits in chicken F_2_ populations crossing between White Recessive Rock (WRR) and Xinghua (XH). Unfortunately, very few reports concerned genetic mechanisms of FTO in fat deposition in broiler.

Chicken transcription factor *PPAR*γ is a key factor in the regulation of abdominal fat deposition (Sato et al., 2004), and it is a master regulator of chicken abdominal adipocyte development *in vitro* (Wang L. et al., 2012; Yu et al., 2014). This transcription factor was detected among the differentially expressed proteins in adipose tissue of divergently selected broilers (Wang D. et al., 2009). Correlation coefficients of the gene expression with AFW and AFP were significantly higher (Larkina et al., 2011). *PPAR*γ expressions were up-regulated with age and significantly higher in fat chicken than lean chicken lines at 2, 3, and 7 wk of age (Sun et al., 2014).

A previous study on broiler adipose tissue development investigated the effect of high-caloric diet vs. standard diet on mRNA expression of 8 candidate genes [*fibroblast growth factor receptor 3* (*FGFR3), EPH receptor B2 (EPHB2), insulin like growth factor binding protein 2* (*IGFBP2*)*, gremlin 1, DAN family BMP antagonist* (*GREM1*)*, tenascin C* (*TNC*)*, collagen type III alpha 1 chain* (*COL3A1*)*, acyl-CoA binding domain containing 7* (*ACBD7*), and *stearoyl-CoA desaturase* (*SCD*)] in abdominal fat at 2, 4, 6, and 8 wk of age. *FGFR3* expression was affected significantly by diet, age, and diet:age interaction; *COL3A expression* was down-regulated by high-caloric diet; expression of *EPHB2, ACBD7*, and *SCD* were affected by age; expression of *TNC* was modulated by age:diet interaction; changes in *GREM1* and *IGFBP2* expressions were statistically similar (Bohannon-Stewart et al., 2014).

The *glucosamine-6-phosphate deaminase 2* (*GNPDA2*) is a member of *Glucosamine-6-phosphate* (*GlcN6P*) *deaminase subfamily*, which encodes an allosteric enzyme of GlcN6P (Ouyang et al., 2016). In humans, the identified SNP rs10938397 of *GNPDA2* gene is found to be associated with adipose tissue accumulation and obesity (Renström et al., 2009). In chicken, *GNPDA2* gene expression has been examined in XH chickens. This gene is highly expressed in chicken abdominal fat, duodenum and hypothalamus, which may relate to fat metabolism and energy balance. One SNP g.6667C>T of this gene shows a significant association with AFW in F_2_ populations crossing between WRR and XH chicken (Ouyang et al., 2016). *GNPDA2* gene expression is altered in response to fasting in XH chicken. The *GNPDA2* gene expression was up-regulated with a high-glucose-fat diet compared to the control basal diet in adipose tissue (abdominal fat and subcutaneous fat) in XH chicken. When chicken *GNPDA2* was overexpressed in abdominal preadipocytes, the mRNA level of some genes involved in fat and/or energy metabolism such as *acetyl-coA carboxylase alpha* (*ACACA*), fatty acid synthase (*FASN*), *FTO*, and *PPARG coactivator 1 alpha* (*PPARGC1A*) were up-regulated, whereas *GNPDA2* knockdown had the opposite effect. These data show that the *GNPDA2* may regulate fat deposition in both human and chicken.

Recently, Jin et al. (2017) *investigated the expression of 6 top candidate genes [steroid 5 alpha-reductase 3 (SRD5A3), sarcoglycan zeta (SGCZ), DLC1 Rho GTPase activating protein (DLC1), 1,4-alpha-glucan branching enzyme 1 (GBE1), polypeptide N-acetylgalactosaminyltransferase 9 (GALNT9), and DnaJ heat shock protein family (Hsp40) member B6 (DNAJB6)]* obtained from GWAS result (unpublished data) in abdominal fat between fat and lean broilers of *NEAUHLF* population. SRD5A3 and *SGCZ* expressions were significantly positively associated with AFW and AFP. *DNAJB6* expression was significantly negatively correlated with AFW and AFP. However, the *DLC1, GBE1*, and *GALNT9* are expressed in abdominal fat but no association with AFW and AFP has been observed. Of these *GBE1* is differentially expressed between fast- and slow-growing chickens (Claire D'Andre et al., 2013), which is expressed at higher level in fast-growing chicken. For the observed effects, it is still unclear how these genes regulating abdominal fat deposition. Using microarray and RNA-seq, many candidate genes involved in abdominal fat deposition in chickens have been identified. These candidate genes will be summarized in a section on gene expression studies.

### Epistatic interactions contributing to abdominal fat deposition

Epistasis is an interaction between two or more genes or their products of mRNA or protein to affect a single trait (Warden et al., 2004), or the product of one gene may inhibit the expression of another gene. The analysis of epistatic relationships between fat and lean lines of chicken abdominal fat trait can suggest ways in which genes control a trait. In chicken, it has been suggested that the epistatic interaction between QTL or among the candidate genes for specific trait could be a useful in defining the genetic architecture of complex traits (Carlborg et al., 2003). The association analysis, pairwise epistasis analysis and chicken 60 K SNP chip have been used as a basic method and tool for detecting epistatic interaction of genes and variants in abdominal fat in chicken (Hu et al., 2010a,b; Li et al., 2013).

A previous work on chicken abdominal fat investigated the epistatic interactions among 10 candidate genes, and constructed a network of the interacting genes (Hu et al., 2010a). The epistatic effects among the 10 candidate genes contributing to phenotypic variation on the AFW were detected in both lines, and the number of significant epistatic effects was much higher in the fat line than in the lean line. Also, Hu et al. (2010b) analyzed the epistatic effect between two SNPs c.2292G>A in *ACACA* and c.-561A>C in *fatty acid binding protein 2* (*FABP2*) on AFW and AFP. The additive × additive epistatic components between these two SNPs were detected to be significant or suggestively significant on both AFW and AFP in lean lines, while, it was not significantly associated with either AFW or AFP in fat lines at 7 wk of age, suggests that the epistasis mode may be dissimilar between the lean and fat chicken lines. In addition, Li et al. (2013) studied the epistatic effects on abdominal fat content in broiler using genome-wide SNP-SNP interaction analysis. Fifty-two pairs of SNPs were identified, comprising 45 pairs showing an additive × additive and 7 pairs showing an additive × dominance epistatic effect. The contribution rates of significant epistatic interactive SNPs ranging from 0.62 to 1.54%, with 47 pairs contributing more than 1%. The SNP-SNP network affecting abdominal fat weight was constructed. The network contains SNPs Gga_rs14303341 and Gga_rs14988623 believed to be the important nodes, and also the interaction between GGAZ and GGA8 was mentioned. 22 QTL, 97 genes, and 50 pathways were annotated on the epistatic interactive SNP-SNP network. These data indicate that the quantitative traits of abdominal fat deposition are often the result of a complex process controlled by multiple loci (genes).

### Copy number variations (CNVs) affecting abdominal fat traits

CNV is a form of genomic structural variation, in which a large DNA segment is either duplicated or deleted (Sudmant et al., 2015). The variation in the DNA segments is approximately ranging from kilobases to megabses (Mb) in size. The detection methods of CNV contain the application of array-based comparative genomic hybridization, SNP microarray and next-generation sequencing. In chicken, CNVs affecting individual genes such as *endothelin-3* gene has been linked to visceral pigment deposition in Silkies (Dorshorst et al., 2011). CNVs have also been found to influence gene expression (Stranger et al., 2007). To our knowledge, only one CNV study of abdominal fat deposition in chicken has been conducted so far. Using the 60 k SNP chip, Zhang H. et al. (2014) identified 188 and 271 CNV regions (CNVRs) in the lean and fat broiler lines, respectively. The fat lines have got less CNVRs than the lean lines, this may be due to differences in the bird numbers (272 vs. 203 in the fat and lean lines, respectively) and selection signature (Zhang et al., 2012b), which mentioned that selective breeding for abdominal fat traits may lead to CNV modifications. Integrated genome analysis of the CNV regions, QTL and selection signature for abdominal fat content has been conducted by the same study. The integrated genome analysis suggests that 14 candidate genes [including *solute carrier family 9 member A3* (*SLC9A3*), *guanine nucleotide binding protein* (*GNAL*; also known as *G protein*), *SPARC/osteonectin, cwcv and kazal like domains proteoglycan 3* (*SPOCK3*), *annexin A10* (*ANXA10*), *IKAROS family zinc finger 2* (*HELIOS*), *myosin light chain kinase* (*MYLK*), *coiled-coil domain containing 14* (*CCDC14*), *sperm associated antigen 9* (*SPAG9*), *SRY-box 5* (*SOX5*), *visinin like 1* (*VSNL1*), *structural maintenance of chromosomes 6* (*SMC6*), *GEN1, Holliday junction 5' flap endonuclease* (*GEN1*), *mesogenin 1* (*MSGN1*), and *zona pellucida protein* (*ZPAX*)] may control abdominal fat deposition. However, whether any of these genes has a critical role in chicken adipogenesis is not known.

### Single nucleotide polymorphisms (SNPs) affecting abdominal fat deposition

SNP is a variation among individuals in a single nucleotide that occurs at a single position based on the known reference genome, where each variation exists in at least 1% of the population. Individual SNPs may be present in one chicken population and absent from another. Most SNPs are detected by direct genomic DNA sequencing, microarray hybridization, and SNP genotyping technologies. By studying these SNPs or DNA polymorphisms in different subpopulations, it may be feasible to define important genetic events and to predict a chicken's susceptibility to diseases. Because of their frequency and distribution throughout the chicken or human genome, SNPs have proven to be valuable genetic markers (Scuteri et al., 2005; Renström et al., 2009; Jia et al., 2012). In this section, the associations of SNPwith abdominal fat traits in chicken will be summarized and discussed. Body weight or BMI may reflect the status of abdominal adipose tissue development or obesity phenomenon, and it has been widely reported in both avian and mammalian studies (Frayling et al., 2007; Gilsing et al., 2012; Jia et al., 2012). However, in this review, we will only summarize the genes or SNPs that significantly associated with abdominal fat traits in chicken. Another topic of practical importance is the finding of short insertions and deletions (Indels). Indels are also commonly referred to as deletion-insertion polymorphisms (DIPs). Sequence variations or DNA polymorphisms in many genes have been found to be significantly associated with chicken abdominal fat traits (see Table 2). These DNA polymorphisms were detected by direct-DNA sequencing of polymerase chain reaction (PCR) products with dye-terminator chemistry analyzed on automated DNA sequencers. SNPs can be located in the different parts of the gene, such as 5′-flanking region, 5′-untranslated region, intron, exon, and 3′-untranslated region (Leng et al., 2009; Nie et al., 2009; Qing et al., 2011; Ouyang et al., 2016). However, SNPs occur in non-coding regions more frequently than in coding regions of the genome (Castle, 2011). In general, the genetic variation at the nucleotide level influences the transcription and translation from gene to protein expression subsequently influences adipose tissue development. A nucleotide substitution that does change the amino acid sequence specified by a codon is named a non-synonymous SNP. A nucleotide substitution that does not change the amino acid sequence specified by a codon is named a synonymous SNP. It has been found that non-synonymous and synonymous coding SNP shared similar likelihood and effect size for disease association (Chen et al., 2010).

**Table 2:** DNA polymorphisms significantly associated with chicken abdominal fat traits.

**Polymorphisms**	**Chr**.	**Gene symbol**	**Abdominal fat traits**	**Population or breed/line (age; days)**	**References**
C2833A	5	*TGFB3*	AFW and AFP	F_2_ Leghorn × Fayoumi (56 d)	Li et al., 2003
9-bp indel	1	*THRSPα*	AFW	Broiler × Leghorn cross (56 d)	Wang et al., 2004
9-bp indel	1	*THRSPα*	AFW	F_2_ WRR × XH (90 d)	d'André et al., 2010
A663T	7	*IGFBP2*	AFW	F_2_ WRR × XH (90 d)	Lei et al., 2005
C1029T	1	*PPARα*	AFW and AFP	Arber Acres broiler (49 d)	Meng et al., 2005
g.-1784_-1768del17	12	*PPAR-γ*	AFW and AFP	NEAUHLF (49 d)	Qing et al., 2011
c.-1241G>A	12				
c.-75G>A	12				
A570C	1	*IGF1*	AFP	F_2_ Leghorn × Fayoumi (56 d)	Zhou et al., 2005
C1032T	7	*IGFBP2*	AFW and AFP	F_2_ NEAURP (84 d)	Li et al., 2006
1196C>A	7	*IGFBP2*	AFW and AFP	F_2_ NEAUHLF (49 d) and F_2_ NEAU (84 d)	Leng et al., 2009
C/T exon 1	2	*FABP4*	AFP	Beijing-YOU (90 d)	Luo et al., 2006
C/T exon 1	2	*FABP4*	AFW and AFP	F_2_ NEAURP (84 d) and F_2_ CAURP (84 d)	Wang et al., 2006
A/G exon 3	2	*FABP4*	AFP	Arbor Acres and Baier chickens (84 d)	Wang Q. et al., 2009
G144762A	27	*GH*	AFW and AFP	F_2_ WRR × XH (90 d)	Lei et al., 2007
c.406T>C	8	*PRKAB2*	AFW	5 pure broiler lines and 3 crossbreeding lines (90 d)	Wang J. L. et al., 2008
C-2047G	12	*GHRL*	AFW	F_2_ WRR × XH (90 d)	Nie et al., 2009
c.782G>A	5	*ATGL*	AFW	F_2_ WRR × XH (90 d)	Nie et al., 2010
c.2292G>A	19	*ACACA*	AFW and AFP	F2 NEAUHLF (49 d) and F2 NEAU (84) d)	Tian et al., 2010
g.240066C>T	13	*ADRB2*	AFP	F_2_ Anak broiler × White leghorn (70 d) and backcrossed population (42 d)	Twito et al., 2011
g.2422364G>A	10	*MFGE8*			
g.2422376G>A	10	*MFGE8*			
rs10731268	28	*ACSBG2*	AFW and AFP	F_2_ WRR × XH (90 d)	Claire D'Andre et al., 2013
rs15248801	28	*ACSBG2*	AFW		
rs15822158	18	*FASN*	AFP		
rs15822181	18	*FASN*	AFW		
C3286>T	9	*GHSR*	AFW and AFP	Arian broiler (42 d)	Darzi et al., 2014
g.7300C>T	11	*KCTD15*	AFW	F_2_ WRR × XH (90 d)	Liang et al., 2015
g.32333C>T	11	*KCTD15*	AFW		
g.6667C>T	4	*GNPDA2*	AFW	F_2_ WRR × XH (90 d)	Ouyang et al., 2016

### Identification of candidate genes associated with abdominal fat traits using genome wide association studies (GWAS)

GWAS is a technique able to screen majority or entire of the genome using dense genomic markers and it has been developed and utilized widely in the analyses of complex traits in both avian and human (Scuteri et al., 2005; Frayling et al., 2007; Sun Y. et al., 2013). GWAS identify SNPs and other variants in genomic DNA associated with specific traits that were not identified by previous microsatellite-based genotyping studies. Using Illumina Bead Array of 3072 SNPs, significant association of 15 and 24 SNPs with abdominal fat percentage in F2 populations crossing between Broiler × Fayoumi or Broiler X Leghorn at 8-wk-old, respectively, has been reported (Abasht and Lamont, 2007). These SNPs are located on 10 chromosomes (GGA1, 2, 3, 4, 7, 8, 10, 12, 15, and 27) and considered to be associated with QTL with cryptic alleles as an essential factor in heterosis for fatness in the two chicken F_2_ populations. The candidate genes identified by Abasht and Lamont, including *FABP4, apolipoprotein B* (*apoB*), *insulin like growth factor 1* (*IGF1*), *insulin-like growth factor 2* (*IGF2*), *L-FABP, Myostatin, PPARGC1A, transforming growth factor beta 3* (*TGFB3*), and *thyroid hormone responsive* α (*THRSP*α). Of these, *PPARGC1A* (also known as *PGC-1*α) is a master regulator of energy metabolism. *PPARGC1A* may be also involved in regulating blood pressure, cholesterol homoeostasis, and the development of obesity (Tobina et al., 2017). It has been demonstrated that myostatin increased expression of CCAAT/enhancer-binding protein alpha (C/EBPα) and adipogenesis in mesenchymal stem cells, suggesting a *myostatin* can in turn modulate *PPAR-*γ expression and/or function, reviewed by Shi et al. (2007). In chicken, studies have shown that DNA polymorphisms in *FABP4, IGF1, TGFB3* and *THRSP*α significantly associated with abdominal fat traits (Li et al., 2003; Wang et al., 2004, 2006; Zhou et al., 2005; Wang Q. et al., 2009). *L-FABP* may also be involved in lipid metabolism (Zhang et al., 2013). Using the Illumina 60 K SNP Beadchip in Beijing-You chicken samples (100-d-old), Liu et al. (2013) identified several candidate genes [*Pumilio homolog 1* (*PUM1*), *small nuclear ribonucleoprotein U5 subunit 40* (*SNRNP40*), *zinc finger protein 521* (*ZNF521)*, and *ankyrin repeat and SOCS box containing 6* (*ASB6*)] associated with abdominal fat traits. To date, it remains unclear the role of these 4 candidate genes in fat deposition.

Sun Y. et al. (2013) used GWAS (600 K SNP array) and mRNA expression analysis to identify loci and genes influencing abdominal fat traits in chicken. They identified SNPs at 15 loci were associated with abdominal fat traits (at day 93 of age) in chicken F_2_ resource population crossing between Beijing-You chickens and Cobb broilers. Seven candidate genes [*RET proto-oncogene* (*RET*)*, natriuretic peptide B* (*NPPB*)*, sterol regulatory element binding transcription factor* (*SREBF*)1*, collagen type XII alpha 1 chain* (*COL12A1*)*, vacuolar protein sorting 4 homolog B* (*VPS4B*)*, BR serine/threonine kinase 2* (*BRSK2*)*, forkhead box C1* (*FOXC1*)] containing or near the SNPs significantly associated with abdominal fat traits were determined. The mRNA expressions of these 7 candidate genes were evaluated by quantitative real-time PCR, expressions of *RET, NPPB* and *SREBF1* were found to be significantly up-regulated in chicken with the highest-abdominal fat content group compared to those in the lowest-abdominal fat group. However, the expressions of *COL12A1, VPS4B, BRSK2*, and *FOXC1* were found to be significantly down-regulated in highest-abdominal fat group or lowest-abdominal fat group, but the exact reason for this is unknown. *RET* (Söhle et al., 2012), *SREBF1* (Sekiya et al., 2007; Sayolsbaixeras et al., 2016), and *NPPB* (Glöde et al., 2017) are known to play a role in lipid metabolism in human or rodents. In chicken, *SREBF1* gene was highly expressed in abdominal adipose tissue (Wang et al., 2010) and is involved in fat metabolism in Beijing-You chickens (Fu et al., 2014). Expression of *NPPB* is positively correlated with the abdominal fat weight of Beijing-You chickens (Huang et al., 2015b).

### Selection signature analysis of abdominal fat traits

Selection signature analysis is a promising approach to address the unexplained variability within a data sample from genetic drift and hitchhiking effect. Studies of selection signature revealed many genes associated with chicken abdominal fat traits. Integrated analysis of selection signature and GWAS provides a new approach that has the powers of both methods. Integrated analysis of selection signature and GWAS (60 k SNP chip) has been reported between lean and fat chicken lines (Zhang et al., 2012a). This integrated analysis identified 10 selection signatures on chromosomes 1, 2, 4, 5, 11, 15, 20, 26, and Z. The *proprotein convertase subtilisin/kexin type 1* (*PCSK1*; also known as *PC1*) region (Z chromosome) at 55.43–56.16 Mb has been identified as the candidate region for abdominal fat yield by the same study. This region had 26 SNP markers and 7 genes [*membrane associated ring-CH-type finger 3* (*MARCH3), solute carrier family 12 member 2SLC12A2, fibrillin 2, endoplasmic reticulum aminopeptidase 1, calpastatin, PCSK1* and *elongation factor for RNA polymerase II 2*], and it is believed to be the most heavily selected region. Genetic variants in the human *PCSK1* gene may be associated with obesity (Renström et al., 2009). To date, it remains unclear whether these 26 SNP markers or the 7 genes have any influence on the regulation of abdominal fat deposition in chicken. Another genome-wide signature analysis was performed between lean and fat chicken lines (Zhang et al., 2012b). This genome-wide signature analysis identified 10 candidate genes [*retinoblastoma 1(RB1), Bardet-Biedl syndrome 7 (BBS7), monoamine oxidase A (MAOA), monoamine oxidase B (MAOB), EH domain binding protein 1 (EHBP1), LRP2 binding protein (LRP2BP), low-density lipoprotein receptor-related protein 1B (LRP1B), myosin VIIA (MYO7A), myosin IXA (MYO9A), and phosphoribosyl pyrophosphate synthetase-associated protein 1 (PRPSAP1)*] may play a role in chicken abdominal fat accumulation.

### Gene expression studies

#### Microarray studies

A growing number of biomedical and biological research-questions count on acquiring global views of gene expression. Microarray technology is a powerful technique used to compare differences in gene expression between two mRNA samples. Gene expression analysis by microarray and high throughput RNA sequencing (RNA-Seq) provides a wide scanning of the transcriptome. Using microarray or high-throughput sequencing in chicken high and low abdominal fat samples, researchers have identified many candidate genes related to abdominal adipose tissue development. (Wang et al., 2007) conducted gene expression analysis (microarray) in abdominal adipose tissue at 7-wk-old between fat and lean chicken lines (NEAUHL populations). The gene expression analysis detected 13,234–16,858 probe sets. Of these, several genes involved in lipid metabolism and immune response, such as *FABP, thyroid hormone-responsive protein* (*Spot14*)*, lipoprotein lipase (LPL), insulin-like growth factor binding protein 7 (IGFBP7)*, and *major histocompatibility complex* (*MHC*) were highly expressed. In contrast, some genes related to lipogenesis, such as *leptin receptor, SREBF1, ApoB*, and *IGF2* were not detected. 230 differentially expressed genes between the two lines in adipose tissue have been found by the same study. These differentially expressed genes are found to be mainly involved in lipid metabolism, signal transduction, energy metabolism, tumorigenesis, and immunity. The most highly expressed genes, such as *A-FABP* and *LPL* were apparently not differentially expressed between the two lines (Wang et al., 2007). In contrast, these two genes (*A-FABP* and *LPL*) have been found to be differentially expressed between high-fat vs. low-fat group, and fast-growing vs. slow-growing lines in chickens (Shi et al., 2010; Claire D'Andre et al., 2013). Fast growing WRR and slow-growing XH chickens were used to characterize specific genes for fat deposition by Affymetrix Genechip® Chicken Genome Arrays 32773 transcripts (Claire D'Andre et al., 2013). 400 genes in the liver and 220 genes in hypothalamus were detected to be differentially expressed in fast-growing and slow-growing chickens, respectively. Expression levels of gene involved in lipid metabolism [*sulfotransferase family cytosolic 1B member 1* (*SULT1B1*), *acyl-CoA synthetase bubblegum family member 2* (*ACSBG2*), *patatin like phospholipase domain containing 3* (*PNPLA3*), *LPL, acyloxyacyl hydrolase* (*AOAH*)], carbohydrate metabolism [*mannosyl (alpha-1,3-)-glycoprotein beta-1,4-N-acetylglucosaminyltransferase, isozyme B* (*MGAT4B*), *xylulokinase* (*XYLB*), *GBE1, phosphoglucomutase 1* (*PGM1*), *hexokinase domain containing 1* (*HKDC1*)], cholesterol biosynthesis [*farnesyl diphosphate synthase* (*FDPS*), *lanosterol synthase (2,3-oxidosqualene-lanosterol cyclase)* (*LSS*), *3-hydroxy-3-methylglutaryl-CoA reductase* (*HMGCR*), *NAD(P) dependent steroid dehydrogenase-like* (*NSDHL*), *24-dehydrocholesterol reductase* (*DHCR24*), *isopentenyl-diphosphate delta isomerase 1* (*IDI1*), *malic enzyme 1*(*ME1*), *hydroxysteroid 17-beta dehydrogenase 7* (*HSD17B7*), and other processes [*cytochrome P450, family 1, subfamily A, polypeptide 1* (*CYP1A4*), *cytochrome P450, family 1, subfamily A, polypeptide 1* (*CYP1A1*), *aldo-keto reductase family 1 member B10-like 1* (*AKR1B1*), *cytochrome P450 family 4 subfamily V member 2* (*CYP4V2*), and *D-aspartate oxidase* (*DDO*)] were higher in the fast-growing chicken than in the slow-growing chickens. Another study by Resnyk et al. (2013) conducted microarray analysis in chicken samples with high or low abdominal fat deposition (2.5-fold difference at 9-wk-old). They revealed that 131 differentially expressed genes as the main effect of genotype, 254 differentially expressed genes as an interaction of age and genotype and 3,195 differentially expressed genes as the main effect of age. Many of these differentially expressed genes belong to different pathways controlling the lipid synthesis, metabolism and transport or endocrine-signaling pathways (adipokines, retinoid and thyroid synthesis). In addition, using Ingenuity Pathway Analysis (IPA), many target genes of the transcription factors that control the divergence of abdominal fatness between fat and lean chickens have been predicted by the same study. For example, *C/EBP*α: 34-; *PPAR*γ: 48-; *SREBF1*: 29-target genes, respectively. However, all these predicted target genes still need to be validated empirically. Moreover, many adipogenic and lipogenic genes are highly expressed in high-abdominal fat chicken, whereas many others involved in blood-coagulation pathway are highly expressed in low-abdominal fat chicken (Resnyk et al., 2013). For example, the lipogenic genes such as *FASN, SCD, SREBF1, SREBF2*, and *THRSP*α are differentially expressed and greater in high-abdominal fat chickens than did low-fat chickens. The high expressions of blood-coagulation genes in low-fat chickens may be due to DNA polymorphisms in the blood-coagulation genes of the lean-chicken that can affect blood coagulation protein levels (de Lange et al., 2001). Adipocytes are a major source of free fatty acids in some mammals, such as dog and cat, whereas liver is widely considered as the major source or active site of *de novo* lipogenesis in avian and human (Leveille et al., 1975; Patel et al., 1975; Stangassinger et al., 1986; Richard et al., 1989; Han et al., 2009). However, it has been suggested that abdominal adipocytes could make a more important contribution to fatty synthesis in avian than previously appreciated (Griffin et al., 1992; Resnyk et al., 2015).

#### RNA sequencing technologies

Currently, high-throughput RNA-Seq techniques have been widely used to uncover molecular biomarkers that may serve as potential new predictors and to examine differential or gene expression level between sample groups. In addition, RNA-Seq is a wider dynamic range and more sensitive than microarrays, with novel detection of transcript and long non-coding RNA (Weikard et al., 2013). RNA-Seq analysis was performed by Resnyk et al. (2015) to identify candidate genes for abdominal fat deposition in two broiler lines which exhibit a 2.8-fold difference in abdominal fatness at 7-wk of age. They showed that about 164 genes were up-regulated in fat chicken, while 155 genes were up-regulated in lean chicken. High expressions of lipogenic, angiogenic, and adipogenic genes were detected in fat chickens, whereas numerous hemostatic and lipolytic genes are expressed higher in lean chicken. The highest expressed and differentially expressed fat metabolism genes including 12 genes [*fatty acid binding protein 3* (*FABP3*), *fatty acid binding protein 5* (*FABP5*), *fatty acid desaturase 2*(*FADS2*), *FASN, glycerol-3-phosphate dehydrogenase 1* (), *hexokinase 2* (), *HSD17B7, IGFBP2, malate dehydrogenase 2* (*MDH2*), *progesterone receptor membrane component 1* (*PGRMC1*), *serine incorporator 1* (*SERINC1)*, and *SCD*] were found to be up-regulated and three genes [*ATP-binding cassette, sub-family A (ABC1), member 1* (*ABCA1*), *IGFBP7*, and *platelet derived growth factor receptor beta* (*PDGFRB*)] were found to be down-regulated. In addition, 21 highest expressed fat metabolism genes including *PPAR*γ, *SREBF2, FABP4, adiponectin, C1Q and collagen domain containing* (*ADIPOQ*), *LPL, GHR, perilipin* (*PLIN*) 1, *PLIN2, signal transducer and activator of transcription 5B* (*STAT5B*), *ELOVL fatty acid elongase 1* (*ELOVL1*), *ACACA, ACACB, ACAD9, acyl-CoA dehydrogenase, long chain* (*ACADL*), *ATP citrate lyase* (*ACLY*), *acyl-CoA oxidase 1* (*ACOX1*), *acyl-CoA synthetase long-chain family member 1* (*ACSL1), acyl-CoA synthetase short-chain family member 2* (*ACSS2*), *ribonuclease A family member k6* (*RNASE6;* also known as *ANG*), *THRSP*α and *NFKB inhibitor alpha* (*NFKBIA*) are found by the same study. However, some well-known fat metabolism genes or transcript isoforms in mammals/birds were not identified, such as *C/EBP*α, *SREBF1, adipose triglyceride lipase* (*ATGL*), and *signal transducer and activator of transcription 3* (*STAT3*) (Rosen et al., 2002; Zimmermann et al., 2004; Lefterova et al., 2008; Resnyk et al., 2013; Yuan et al., 2017). These discrepancies in the expression are probably due to difference in species, age, environmental factors and the stage of adipogenesis. Another integrated analysis of mRNA and miRNA, performed RNA-seq analysis between high and low abdominal fat libraries, a total of 303 differentially expressed genes were detected, and among them 11 genes are involved in fat metabolism-signaling pathways (*ATP binding cassette subfamily D member 3* (*ABCD3*), *FADS2, SCD, PECR, AKT serine/threonine kinase 1* (*AKT1*), and *caveolin 2* (*CAV2*) are upreguated, whereas *ACSL1, chondroadherin* (*CHAD*), *integrin subunit alpha 11* (*ITGA11*) and *laminin subunit alpha 2* (*LAMA2*) are downregulated (Huang et al., 2015a). More recently, another integrated analysis of mRNA and lncRNA, conducted RNA-seq in chicken abdominal preadipocytes at different stages (day 0, 2, 4, and 6) during abdominal preadipocyte differentiation. This RNA-seq identified 1759 genes to be differentially expressed across various stages; days 0, 2, 4, and 6 of preadpiocyte differentiation (Zhang et al., 2017). These differentially expressed genes showed a decline in the number as the differentiation of preadipocytes advanced. Several pathways have been found for the first time by the same study, including the propanoate metabolism, fatty acid metabolism, and oxidative phosphorylation pathways. In summary, the chicken *HSD17B7* gene was detected by microarray and RNA-seq from two studies (Claire D'Andre et al., 2013; Resnyk et al., 2015) to be highly expressed in fast-growing chicken or high-fat group; therefore, it may play a potential role in fat deposition in chicken.

### Multiple factors regulating abdominal adipocyte precursor cell proliferation and differentiation *in vitro*

Preadipocyte (also known as adipocyte precursor cell) is an undifferentiated fibroblast that can be stimulated by different ways to form an adipocyte. *In vitro* studies of preadipocyte development prepared from abdominal adipose tissue might help us to explore the molecular mechanisms underlying abdominal adipose accumulation because many genes, transcription factors, non-coding RNAs are expressed in tissue/cell-specific manner. Indeed, it is essential to fully identify the genes or pathways that regulated in abdominal fat through culture system, and establish transcriptome atlas for abdominal adipose tissue development.

Adipogenesis is a process in which an undifferentiated mesenchymal precursor differentiates into a preadipocyte, which then undergoes a secondary differentiation stage to become a mature adipocyte. It has been reported that adipogenesis can be completed by various stages, including mesenchymal precursor (proliferation; capable of differentiating), committed preadipocyte (proliferation, commitment to differentiate), growth-arrested preadipocyte (loss of proliferation), mitotic clonal expansion (some rounds of cell divisions), terminal differentiation (arrest of cell cycle; *PPAR*γ and *C/EBP*α induction) and mature adipocyte (lipid-filled adipocyte; adipocyte genes transcriptional activation; elevated expression of adipocyte genes), respectively (Gregoire et al., 1998; Sarjeant and Stephens, 2012).

*In vitro* primary cell culture system from the stromal-vascular fraction of chicken abdominal fat has been developed by Ramsay and Rosebrough (2003). Unfortunately, the effect of genes or transcriptional factors on the regulation of preadipocyte proliferation and differentiation has not been well-studied in the chicken, probably because of the lack of chicken preadipocyte cell line. Recently, immortalized preadipocytes were successfully produced from chicken abdominal adipose tissue of 10-day-old (Wang et al., 2007). Both primary adipocyte precursor cells and immortalized cell lines can be used to characterize genetic factors that promote/inhibit preadipocyte proliferation and/or differentiation. However, immortalized cells can modify the biology and the genetic material of the cell and must be taken into consideration in any analysis because these cells have undergone significant mutations to turn into immortal (Marx, 2014). At the cellular level, adipocyte precursor cell proliferation and differentiation are under the regulation of many genes and/or transcription factors, and endocrine factors.

The transcription factor *PPAR*γ is known as the master regulator of adipocyte development in mammals and birds (Tontonoz et al., 1995; Gerhold et al., 2002; Meng et al., 2005; Tontonoz and Spiegelman, 2008; Wang Y. et al., 2008; Yu et al., 2014). C/EBPα is another adipogenic transcription factor and is a key regulator for adipogenesis in mammalian and avian (MacDougald and Lane, 1995; Yu et al., 2014). Terminal adipocyte differentiation is tightly controlled by multiple transcription factors, such as *PPAR*γ and *C/EBP*α, which are up-regulated in a coordinated fashion and could lead to adipocyte-specific gene expression (Rosen et al., 2002). In chicken, it have been reported that *solute carrier family 27 member 1* also known as *FATP1* (Qi et al., 2013), *KLF 2* (Zhang Z. W. et al., 2014), and *PPAR*γ and *C/EBP*α (Yu et al., 2014) controls fat deposition *in vitro*; however, more studies are necessary to clarify the mechanism(s) of action. In mammals, it's well-known that the *GATA binding protein* (*GATA*) *2 and 3* have negative effects on adipogenesis (Sarjeant and Stephens, 2012). In chicken, *KLF2* up-regulates *GATA2* expression (Zhang Z. W. et al., 2014); therefore, both *GATA2* and *GATA3* in chickens may have a similar function to that in mammals.

Several signaling pathways have been reported to be associated with chicken adipogenesis. Among them, adiponectin suppressed chicken preadipocyte differentiation by down-regulating C/EBPα and FASN protein expression, while upregulating ATGL protein expression. The mechanism of action was revealed in which this adiponectin induces p38 mitogen-activated protein kinase (p38 MAPK; also known as MAPK) and activating ATF-2, which leads to inhibition of the TOR/p70 S6 Kinase signaling pathway (Yan et al., 2013). Another signaling pathway, recombinant globular adiponectin inhibits fat deposition by down-regulating adipogenic marker genes, such as C/EBPα and FASN expression, while upregulating ATGL expression in chicken adipocytes. The mechanism of action was explained in which the recombinant globular adiponectin activating the phosphorylation levels of p38 MAPK/ATF-2, while suppressing the phosphorylation levels of TOR/p70 S6 kinase pathways (Jun et al., 2014). Recently, using deep RNA-seq combined with Gene Ontology analysis in the integrated analysis of mRNAs and lnRNAs, Zhang et al. (2017) identified several pathways associated with preadipocyte differentiation at different stages, such as glycerolipid metabolism, mammalian target of rapamycin (mTOR) signaling, PPAR signaling, and MAPK signaling pathways. These pathways are well-known in other species to play essential roles in preadipocyte differentiation. Among them, four signaling pathways are found to be significantly associated with abdominal preadipocyte differentiation, such as Wnt, MAPK, and TGF-b. Furthermore, other novel pathways associated with preadipocyte differentiation were also identified by the same study, such as propanoate metabolism, fatty acid metabolism, and oxidative phosphorylation.

### MicroRNAs (miRNAs) involved in abdominal fat deposition

miRNAs are small non-coding single-stranded RNA molecules (found in plants, animals and some viruses) consisting of about 22 nucleotides in length that bind or target the 3′-untranslated region of mRNAs to regulate gene expression post-transcriptionally (Ambros, 2004; Bartel, 2004). miRNAs are produced in the nucleus from precursor molecules termed primiRNAs, which may consist of hundreds or thousands of nucleotides that produce the transcriptional units of polycistronic or monocistronic. miRNAs can affect protein production by translational repression or mRNAs destabilization (Filipowicz et al., 2008; Rottiers and Naar, 2012). miRNAs regulate a wide range of biological processes, including adipogenesis, lipid metabolism, cell proliferation & differentiation, growth, apoptosis, carcinogenesis and disease. Early molecular cloning experiment has only identified 47 known miRNAs in chicken abdominal adipose tissue (Wang X. G. et al., 2012). Yao et al. (2011) detected 159 known miRNAs by Solexa sequencing in Arbor Acres preadipocytes isolated from abdominal fat. Recently, using deep sequencing, Wang et al. (2015) identified 33 differentially expressed miRNA in preadipocyte isolated from abdominal adipose tissues of two broiler lines selected for abdominal fat content. The most abundant miRNAs are the let-7 family, suggesting their functional roles in fat accumulation. More recently, integrated analysis of differentially expressed miRNAs and mRNAs has been assessed by deep sequencing in abdominal adipose tissues derived from a cross between a slow-growing (low-abdominal fat) Chinese local breed (Beijing-You) and a rapid-growing (high-abdominal fat) commercial broiler line (Cobb-Vantress). Compared with the low-abdominal fat, 62 differentially expressed miRNAs and 303 differentially expressed genes were determined in the high-abdominal fat group, and 106 differentially expressed genes were predicted to be as a target for the 62 differentially expressed miRNAs (Huang et al., 2015a). Several miRNAs have been predicted to be highly involved in the regulation of chicken abdominal fat or fat cell development; and are presented in Table 3. Of these, seven miRNAs (miR-30d, miR-26a, let7c, let7j, let7f, let7b, and let7a) might be the most involved miRNAs in fat deposition. Further studies should first focus on the mechanisms of action of these seven miRNAs in adipogenesis because they were detected by more than one study to be highly regulated in abdominal adipose tissue development. In chicken, miR-122 predicted to bind *PPAR*γ, suggesting that it may play an essential role in the growth of adipose tissue. gga-miR-19b-3p is a direct target of *ACSL1* by downregulating *ACSL1* gene expression (Huang et al., 2015a). Overexpression of gga-miR-19b-3p promotes both preadipocyte proliferation and their subsequent differentiation in chicken, indicating their functional roles in adipogenesis and fat deposition.

**Table 3:** Several miRNAs found to be highly involved in the regulation of chicken abdominal fat deposition or fat cell accumulation identified by deep sequencing.

**miRNAs**	**Expression or biological status (candidate)**	**Abdominal fat Tissue/cell**	**References**
miR-222, miR-30d, miR-26a, let7c, let7a, let7d, let7j, let7f, let7b, and miR-30a-5p.	Highly expressed	Abdominal preadipocyte	Yao et al., 2011
let-7 miRNA family (let-7a, j, b, f, c, and k)	Most abundant	Abdominal preadipocyte	Wang et al., 2015
miR-206, miR-31, miR-3535, miR-17-3p, miR-429 and miR-200b (up-regulated in fat line), or miR-454 and miR-1b (down-regulated in lean line).	Most significantly differentially expressed		
miR-19a-3p, miR-19b-3p, miR-17-5p, miR-30d, miR-26a, miR-103-3p, miR-27b-3p, miR-142-3p, and miR-92-3p.	Strong candidate	Abdominal fat tissue	Huang et al., 2015a
miR-3535, miR-30e-5p, miR-301b-3p, miR-215-5p, miR-200a-3p, miR-133a-3p, miR-133c-3p, and miR-146b-5p.	Potentially candidate		

### Regulation of abdominal fat deposition by long non-coding RNAs (LncRNAs)

LncRNAs are a novel class of regulatory RNAs with sizes ranging from 200 bp to a greater than 100 kb (Novikova et al., 2013). LncRNAs are involved in various aspects of cell biology such as adipogenesis, hepatic lipid metabolism, energy balance, cell proliferation, cell migration, tumor metastasis, transcriptional interference, activation, post-transcriptional regulation, genomic imprinting, X chromosome inactivation, nuclear trafficking, and chromatin modifications (Ma et al., 2013; Sun L. et al., 2013; Tripathi et al., 2013; Maass et al., 2014; Zhao and Lin, 2015; Li et al., 2016). As far as we know, only one lncRNA study in abdominal preadipocytes in chicken has been reported to date (Zhang et al., 2017). This lncRNA study identified 27,023 novel lncRNAs, 1,336 differentially expressed lncRNAs, and 1,759 differentially expressed mRNA in preadipocytes at different stages (day 0, 2, 4, and 6) of differentiation. The number of differentially expressed genes (lncRNAs and mRNAs) across different stages declined as differentiation advanced. In addition, the identified novel lncRNAs in chicken abdominal preadipocytes shared many properties with those identified in other species but fewer in the number of exons and shorter in the sequence size. 4915 target protein-coding genes were identified by the same study. Furthermore, among differentially expressed lncRNAs and mRNAs, 9 central and highly connected lncRNAs and mRNAs [XLOC_068731, XLOC_022661, XLOC_045161, XLOC_070302, *chromodomain helicase DNA binding protein 6* (*CHD6*), *LLGL1, scribble cell polarity complex component* (*LLGL1*), *neuralized E3 ubiquitin protein ligase 1B* (*NEURL1B*), *kelch like family member 38* (*KLHL38*), and *ARP6 actin-related protein 6 homolog* (*ACTR6*)] have been identified by bioinformatic analyses (Cytoscape 3.4.0 software). The identification of these connections important because studies have proposed that lncRNAs could control and are highly correlated with the expression of adjacent or neighboring mRNAs (Ren et al., 2016; Wang et al., 2016), suggesting that lncRNAs may act in cis on neighboring protein-coding genes to control the differentiation of abdominal preadipocyte.

### Epigenetic alteration controls abdominal fat traits

Epigenetic mechanisms are likely to be involved in the development of abdominal adipose tissue. During adipose tissue development, epigenetic regulators are capable of promoting the transcription of a selective group of gene and participate in adipogenesis (Li et al., 2010; Musri and Parrizas, 2012). Gene expression during adipogenesis can also be regulated by epigenetic modification, such as DNA methylation. DNA methylation is a key epigenetic contributor to the maintenance of gene silencing. DNA methylation levels in mammals are reprogrammed during development. In avian and mammal, three DNA methyltransferase genes including *Dnmt1, Dnmt3a*, and *Dnmt3b*, coordinately are regulating the methylation of DNA in the genome. *Dnmt1* plays a main role in the maintenance of DNA methylation. On the other hand, *Dnmt3a* and *Dnmt3b* are developmentally regulated genes required for the initiation of *de novo* methylation in chicken and mouse embryos (Okano et al., 1999; Rengaraj et al., 2011). In rodents, maternal supply of methyl groups throughout pregnancy altered the DNA methylation of candidate genes such as *PPAR*α in pups (Lillycrop et al., 2005). It seems that DNA methylation regulates the expression of PPARγ in mammalian and avian adipogenesis (Fujiki et al., 2009; Sun et al., 2014; Yu et al., 2014). In chicken preadipocyte, the CpG loci of *PPAR*γ promoter were highly methylated *in vitro* (Yu et al., 2014). In addition, the promoter methylation status of the chicken *PPAR*γ gene has been investigated by Sun et al. (2014) in NEAUHLF chicken populations; the abdominal fat percentage of the fat chicken line was 4.5-fold higher than that of the lean chicken line. There is a differential methylation between the lean and fat chickens, *PPAR*γ promoter methylation levels were significantly higher in lean than fat broilers, but the mRNA level was in opposite manner at 2, 3, and 7 wk of age during adipose tissue development. Although strong DNA methylation at promoters is well-known to be associated with transcriptional repression, recent studies suggest that DNA methylation outside promoters also plays pivotal roles in gene regulation (Lou et al., 2014).

### Knowledge gaps to be addressed

Abdominal fat deposition is a complex trait and the difficulty this poses in identifying all the interacting genetic, epigenetic and environmental factors involved. Although results from research on the genetic mechanism of fat deposition in chicken is beginning to accumulate, many gaps in the knowledge base need to be addressed before evidence-based recommendations can be formulated specifically for reducing fat deposition. The focus of abdominal fat deposition research to date has largely focused on detecting the candidate genes by SNPs, GWAS and gene expression studies; however the knowledge gaps are addressed in the following lines: **A**- Circular RNAs (circRNAs) and DNA methylation by whole-genome bisulfite sequencing (WGBS) in abdominal fat deposition need to be identified because they may regulate fat storage. The circRNAs are a new class of non-coding RNA molecules which do not have the terminal structures (5-cap and polyA tail) but are covalently linked to form a closed circular structure. CircRNAs are widely expressed in animal cells and have been shown to act as sponges for miRNAs (Hansen et al., 2013). Circ-ZNF609 is a circular RNA that can be translated into a protein, and mediate transcriptional and post-transcriptional control of gene and protein expression (Legnini et al., 2017). The WGBS is highly sensitive and provides quantitative DNA methylation measurements (Lou et al., 2014). **B**- Data are also needed on the functional mechanism and roles of the many detected candidates from genes, transcription factors, pathways, miRNAs, and lncRNAs to identify novel biomarkers for reducing abdominal fat accumulation. **C**- Research is needed to determine the regulatory function of DNA methylation of gene promoters, miRNAs, lncRNAs, and circRNAs for chicken abdominal fat deposition or adipogenesis. In addition, analyzing the interactions of mRNAs, miRNAs, lncRNAs and circRNAs should be a priority for further research. **D**- Additional GWAS at different-chicken developmental stages in abdominal fat between fat and lean broiler lines of different genetic backgrounds will also be required. **E**- For DNA polymorphism, considering that SNP alleles (Table 2) had a useful effect on reducing abdominal fat deposition, it would be possible to do marker-assisted selection programs favoring the alleles for reducing the abdominal fat-deposition in chickens; this hypothesis must be evaluated in selection programs. To do the selection programs applicable, it would be important to further analyze the effects of these gene-polymorphisms by using populations of different genetic backgrounds (different breeds and lines) and with a large population size. **F**- In mammals, many of current genetic dissections are devoted to the investigation of pathways regulated in important biological processes such as fat metabolism and growth. Examining the epistatic relationships among genes can aid to sort out the role that each gene plays in these processes. Additional epistatic relationships are required because studying the epistatic relationships among genes can help to sort out the role that each gene plays in the fat deposition. **G**- Future studies are needed to investigate the existence of BAT and beige adipocytes in chicken.

## Conclusions

Numerous studies have been performed to identify associations between the genetic and abdominal fat deposition. The chicken abdominal fat deposition is a complex and orchestrated by induction of many genes and molecular components. With the development of biotechnology and bioinformatics, approaches such as QTL analysis, selection signature analysis, candidate gene approach, CNVs, epistatic interaction of genes and variants, SNP, GWAS, microarray, RNA-seq, and miRNA high-throughput sequencing can be used to identify genetic factors involved in the abdominal fat deposition. Using these approaches, many important candidates from multiple genetic factors including genes, transcription factors, signaling pathways, miRNAs, and IncRNAs have been identified. However, very few studies have investigated in-depth the functional role of the identified important candidates. Hence, future investigations should focus on further establishing the functional roles and links between the identified important candidates and abdominal fat deposition; and demonstrate the underlying molecular mechanisms. In-depth knowledge of the regulatory roles and molecular mechanisms of the identified important candidates during *in vivo* abdominal fat development and/or during *in vitro* proliferation and differentiation of preadipocytes may help to identify novel biomarkers for reducing fat accumulation in chicken or may be as novel therapeutic targets for obesity in human.

## Author contributions

BA conceived, designed, and wrote the paper. JC participated in the critical revising. QN participated in designing, revising and coordination. XZ participated in the design of the paper. All authors reviewed and approved the final version.

### Conflict of interest statement

The authors declare that the research was conducted in the absence of any commercial or financial relationships that could be construed as a potential conflict of interest.

## Abbreviations

ACSBG2: acyl-CoA synthetase bubblegum family member 2
ACSL1: acyl-CoA synthetase long-chain family member 1
ADRB2: beta-2 adrenergic receptor
AFP: abdominal fat percentage
AFW: abdominal fat weight
ATGL: adipose triglyceride lipase
CAURP: China Agricultural University Resource Population
cM: Cri-Map
F_2_: an F_2_ resource population
FABP4: fatty acid binding protein 4
GH: growth hormone
GHR: growth hormone receptor
GHRL: ghrelin/obestatin prepropeptide
GHSR: growth hormone secretagogue receptor
IGF1: insulin like growth factor 1
IGFBP: insulin-like growth factor binding protein
KCTD15: potassium channel tetramerization domain containing 15
LEPR: leptin receptor
NEAU: Northeast Agricultural University
NEAUF2: Northeast Agricultural University F_2_ resource population
NEAUHLF: Northeast Agricultural University high and low fat
NEAURP: Northeast Agricultural University Resource Population
MFGE8: milk fat globule-EGF factor 8 protein
PRKAB2, protein kinase, AMP-activated: beta 2 non-catalytic subunit
TGFB3: transforming growth factor beta 3
wk: week
WPR: White Plymouth Rock
WRR: White Recessive Rock
XH: Xinghua.
